# Melanoma metastasis to the spleen: Laparoscopic approach

**DOI:** 10.4103/0972-9941.51316

**Published:** 2009

**Authors:** Manoel Roberto Maciel Trindade, Rodrigo Blaya, Eduardo Neubarth Trindade

**Affiliations:** Department of Surgery, School of Medicine, Universidade Federal do Rio Grande do Sul, UFRGS and Division of Digestive Surgery, Hospital de Clínicas de Porto Alegre, HCPA

**Keywords:** Melanoma, metastasis, spleen

## Abstract

We report a case of minimally invasive surgery in the management of metastasis to the spleen. A 67-year-old male patient with possible splenic soft tissue melanoma metastasis was referred to our hospital. He had a history of an excised soft tissue melanoma from his back eight months earlier, and the control abdominal computer tomography (CT) scan revealed a hypodense spleen lesion. The patient underwent laparoscopic surgery to diagnose and treat the splenic lesion. The splenectomy was performed and the histological examination revealed a melanoma. The patient had a good postoperative course and was discharged on the second postoperative day. On his 12-month follow-up there was no sign of recurrence. The laparoscopic approach is a safe and effective alternative for treatment of splenic metastases.

## INTRODUCTION

Metastasis to the spleen is considered a rare event in the late course of malignant solid tumors. Most splenic metastases from melanoma are identified at autopsy. In life, they are commonly part of a widespread, end-stage disease and are traditionally treated with systemic chemotherapy regimens. Nevertheless, there are many reports of long-term survival after aggressive surgical treatment of stage IV metastatic melanoma. Some patients with metastatic melanoma of the spleen have a good performance status and no evidence of disease elsewhere. Surgical resection can be a realistic therapeutic option in these cases.[1]

Laparoscopic splenectomy (LS) has rapidly become the surgical approach of choice for patients who require elective splenectomy in the treatment of hematologic disorders.[2] Recent reports have described the use of LS for other malignant disorders. In this article we report the case of a man with a solitary melanoma metastasis that was managed successfully with LS.

## CASE REPORT

A 67-year-old Caucasian male, with a medical history of hypertension, using hydrochlorothiazide and captopril, presented with a mass in his back that had been growing for three years. The mass had been excised and revealed a soft tissue melanoma (clear cell sarcoma). Two months later, the physical examination revealed an enlarged lymph node in the right inguinal area. The patient was then submitted for an inguinal lymphadenectomy that confirmed a metastatic melanoma lesion.

**Figure 1:**
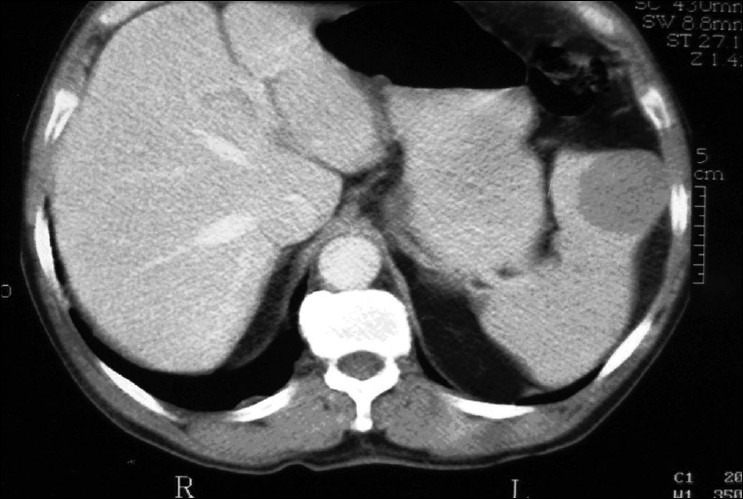
Computer tomography of the abdomen showing a hypodense spleen lesion

Eight months later the control abdominal CT scan revealed a hypodense spleen lesion, measuring 2.5 cm. The lesion was re-evaluated two months later. It had increased to 4.5 cm [Figure 1]. Subsequently, the patient underwent LS to diagnose and treat the splenic lesion.

**Figure 2:**
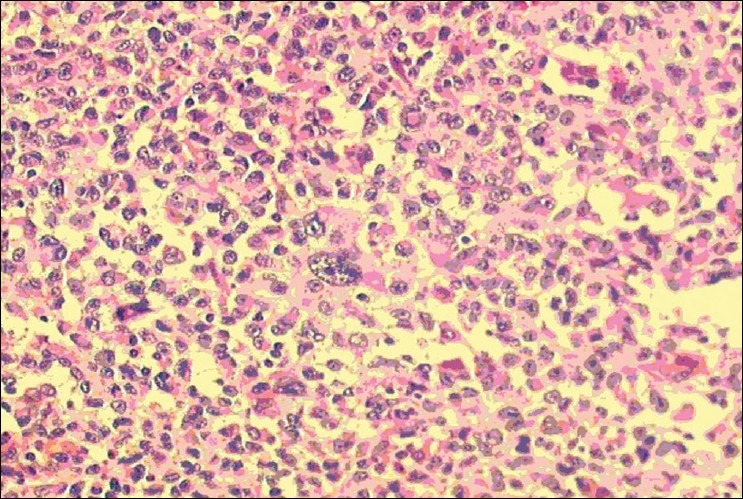
Microscopic photography of the spleen showing a metastatic melanoma (clear cell sarcoma), (H & E, ×200)

The patient was placed in the lateral decubitus position and four subcostal ports were utilized. No other pathological lesions were seen in the abdomen and there was no contiguous involvement of other visceral organs. The splenectomy was completed and the specimen was placed in a leakage-proof bag. The bag was exteriorized to the abdominal wall. An anatopathological analysis confirmed a metastatic lesion of melanoma [Figure 2].

The patient made a good postoperative recovery, started diet on the first postoperative day, and was discharged on the second postoperative day. The patient has completed six months of follow up, without signs of disease recurrence.

## DISCUSSION

Clear cell sarcoma (CCS) or malignant melanoma of soft parts, for many years, was due to the occasional presence of melanin pigment and melanosomes; CCS was thought to be histogenetically related to malignant melanoma. However, CCS bears only a phenotypic resemblance to malignant melanoma. Subsequent genetic studies have demonstrated a characteristic translocation [t (12;22)(q13;q13)], which results in a unique fusion gene that is not found in cutaneous or uveal malignant melanomas, but is instead related to those found in some sarcomas.[3] It is a slow-growing tumor, often with a long duration from the initial symptom to diagnosis average of two years.[4]

The course of CCS is variable and unpredictable. Despite treatment, the overall prognosis is poor and the subsequent widespread dissemination of the disease causes the death of patients. The five-year survival rate with radical excision and adjuvant chemotherapy and/or radiotherapy ranged from 60 to 67% and the 10-year survival rate is about 33%. The most common sites of metastases are the regional lymph nodes and the lungs and less commonly skin, bones, liver, heart, and brain. Sixty to seventy percent of patients with CCS develop metastases within a mean of 18 months to six years.[6]

Splenic metastases from most types of tumor are rare, but they occur more frequently in patients with breast cancer, lung cancer, and melanoma. The majority of splenic metastases from melanoma are identified at autopsy. In life, they are commonly part of a widespread, end-stage disease.[1]

Development of distant metastases in melanoma patients portends a poor prognosis. The median survival of patients with American Joint Committee on Cancer (AJCC) stage IV melanoma is four to eight months. The majority of patients with distant melanoma metastases are treated with chemotherapy, radiotherapy or both. Unfortunately, the response rates to these modalities are low and seem to have little effect on survival.[7]

Nevertheless, there are reports of long-term survival after aggressive surgical treatment for stage IV metastatic melanoma. Surgery for apparently isolated pulmonary metastases, gastrointestinal metastases, and metastases in other visceral sites such as the adrenal gland has been associated with five-year survival rates of 20 to 40%. Even for patients with recurrent stage IV melanoma, prolonged survival after surgery has been reported in a selected group of patients.[8]

In a report of 15 cases with spleen melanoma metastasis treated surgically, survival of seven patients who underwent surgery, with an intention to cure, was 20 months, with two patients living 29 and 30 months after splenectomy, without signs of recurrence.[1]

Laparoscopic splenectomy is the preferred surgical approach for benign hematologic disorders when elective removal of the spleen is indicated. Technical success, minimal morbidity, reduced disability, and high patient acceptance have justified its application. Laparoscopic splenectomy can be performed, with good results, on patients with a variety of malignant hematologic disorders as well. The laparoscopic approach is a safe and effective treatment for splenic melanoma metastases.
